# Evaluation of the feasibility of a video-transmitted surgical ward round: a proof of concept study

**DOI:** 10.1186/s12909-023-04656-9

**Published:** 2023-09-21

**Authors:** Jonas Johannink, Steffen Axt, Alfred Königsrainer, Teresa Festl-Wietek, Stephan Zipfel, Anne Herrmann-Werner

**Affiliations:** 1 Department of General, Visceral and Transplant Surgery, Tübingen University Hospital, Hoppe-Seyler-Str. 3, 72076 Tübingen, Germany; 2 Tübingen Institute for Medical Education (TIME), Medical Faculty of Tübingen, University of Tübingen, Tübingen, Elfriede-Aulhorn-Straße 10, Tübingen, 72076 Germany; 3 Department of Psychosomatic Medicine and Psychotherapy, Tübingen University Hospital, Osianderstraße 5, 72076 Tübingen, Germany

**Keywords:** Surgical ward round, Video based teaching, Remote teaching, Surgical competencies, Interactive seminars, Interprofessional training, Interdisciplinary education

## Abstract

**Background:**

Surgical ward rounds are key element to point-of-care interprofessional postoperative treatment and technical and communicational aspects are relevant for the patient’s safety and satisfaction. Due to COVID-19 restrictions, the training opportunity of experiencing a face-to-face surgical ward round was massively hampered and thus, we developed a digital concept. This study aims to investigate the feasibility of video-transmitted ward rounds integrating surgical and communicational aspects with live streaming from wards. Further, medical students were asked for their satisfaction and their subjective learning success.

**Methods:**

The proof-of-concept study consisted of self-reported subjective evaluation of competences in ward round skills. Qualitative feedback was collected to gain deeper insight and students’ empathy was rated by using the student version of the Jefferson Empathy Scale (JES).

**Results:**

One hundred three medical students participated. The students were satisfied with the video-transmitted ward round (M = 3.54; SD = 1.22). In the subjective evaluation students’ ward round competencies rose significantly (*p* < .001, M_pre_ = 3.00, SD = 0.77; M_post_ = 3.76, SD = 0.75). The surgeon was rated as empathic (M = 119.05; SD = 10.09). In the qualitative feedback they named helpful aspects like including an expert for communication. However, they preferred the face-to-face setting in comparison to the digital concept.

**Conclusions:**

It was feasible to implement a video-transmitted ward round within a pandemic. The format worked technically, was well-accepted and also led to a subjective rise in the students’ competencies. Video-transmitted ward rounds may be integrated to support the medical education, though, they cannot replace the face-to-face setting.

## Introduction

Surgical ward rounds are key element to point-of-care interprofessional postoperative treatment of surgical patients, which warrants central importance in medical education, too [1]. Although ward round teaching is important in every clinical discipline, particularly in surgery specific training is needed to provide a well-structured and focused yet complete physical examination [2–4]. Besides the technical aspects a surgical ward round also contains aspects of communication and interaction pivotal to effective and safe patient care as well as patient satisfaction [5–9]. This is also mirrored in the surgical assessment tool (SWAT) that includes non-technical ward round skills like communication and teamwork as important pillars for effective ward rounds in surgery [10]. As consultants may be unaware of the communicational learning opportunities, efforts to explicitly integrate communication aspects into surgical ward round teaching should be made [11].

Due to COVID-19 restrictions, the training opportunity of experiencing a face-to-face surgical ward round was massively hampered since summer term 2020 as students were not allowed any direct patient contact in teaching [12, 13]. On the other hand, the pandemic was a massive catalyst for the development and implementation of digital learning resources worldwide [14]. Most of these relied heavily on digital communication methods enabled by IP-based tools like videoconferencing software or learning platforms [15]. Clinical educators tried to counterbalance the loss of direct patient contact by specific virtual teaching sessions including display of treatment of COVID-19 patients or correct handling of personal protective equipment (PPE) on wards [16–18]. Also, as several specialties were pretty strained with care for COVID-19 patients and couldn’t offer specific standalone teaching, students were integrated into regular telemedical treatment sessions and learned in kind of a virtual bedside teaching environment [19]. There have also been a variety of attempts to specifically create virtual ward round settings. Glasgow and colleagues for example modified a usual surgical multi-professional ward round: only the attending surgeon was in the room with the patient wearing a hands-free headset whilst the rest of the team were attending online [20]. The majority of the team members and the patients considered it a good substitute in a pandemic. However, as this study included no medical students such a setting might only work for persons already trained in and familiar with the subject. Another study group tried a mixed reality version of a surgical ward round with the physician wearing a HoloLens 2™ whilst in the patient’s room [21]. Although this concept was evaluated as feasible, well-accepted and effective, it only included 11 students in the study which limits generalisability. Also, via the HoloLens 2™ it only allowed an interaction with the surgeon but not with the patient, which might prevent successful active learner involvement deemed so necessary in effective workplace-based learning [22, 23]. Additionally, it didn’t use the normal ward round setting of a surgical ward but specifically taught two selected patients in 60 min making it more a profound kind of bedside teaching than an actual surgical ward round.

Thus, to the best of our knowledge, there hasn’t been any attempt to preserve the traditional surgical ward round to teach medical students this complex skill in a time where direct patient contact was prohibited.

At our surgical department, teaching started as a complete virtual course with asynchronous and synchronous elements but without any patient contact. In 2021, at least seminar teaching was allowed in presence although direct face-to-face patient contacts were still prohibited. We thus created a video-transmitted ward round concept including surgical and communicational aspects of ward rounds with live streaming from wards. With this study, we wanted to prove that such a setting was feasible to perform, worked in its hybrid form, was well-accepted by students, and showed a positive subjective learning effect regarding key elements of ward round competencies.

## Materials

### Study design

We conducted a proof-of-concept study with self-reported evaluation of the acquired competences in ward round skills as well as satisfaction with its implementation. Additionally, we collected qualitative feedback to gain deeper insight. Furthermore, we examined the physician–patient interaction using the Jefferson Empathy Scale.

### Sample

In total, 103 Students participated in the study. Of these were 69.9% female and 30.1% male which corresponds to the general gender relation at the Medical Faculty of Tübingen. 92.9% of the students were in the 9. Semester forming the regular target group while 2.9% and 3.9% were in the 8^th^ and 10^th^ semester taking part in the course earlier or later than regularly scheduled. Eligibility criteria were being medical students attending courses of clinical examination and understanding German.

### Teaching procedure

**Fig. 1:**
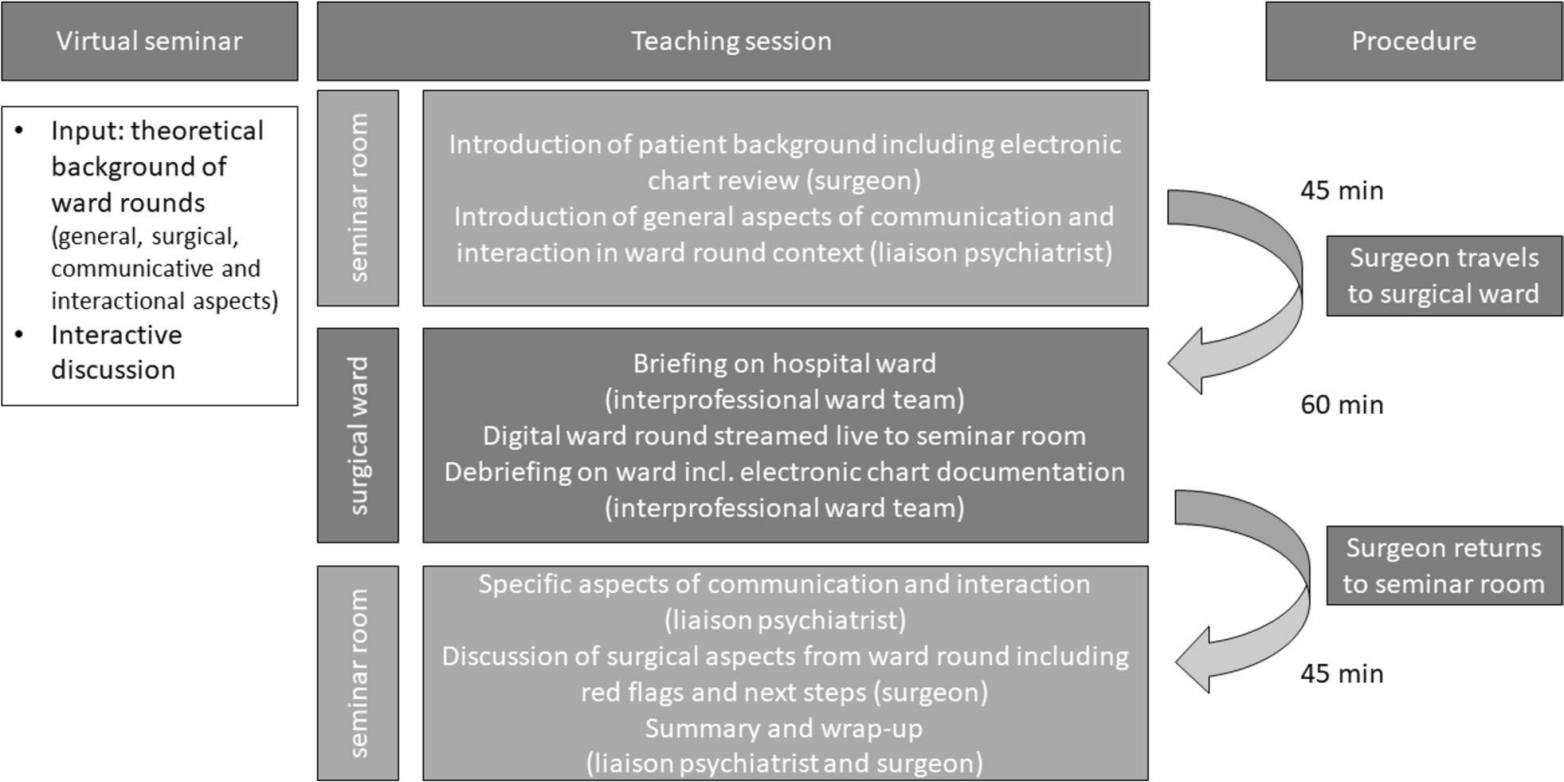
Video-transmittedl ward round teaching elements

The ward round teaching took place within the regular surgical class in year 5 on a weekly basis. On the introductory day, there was a 45min seminar on theoretical background followed by the practical teaching on day 4. It lasted 150min and was taught as a face-to-face seminar by one surgeon and one liaison psychiatrist to reflect on technical as well as non-technical skills within a ward round. The corresponding ward round streamed to the students was performed on a regular ward of the department of visceral surgery, University Hospital Tübingen, Germany. The ward round team included the class surgeon and a nurse, as well as potentially other health care professionals and/or nurses in training or final-year medical students. The liaison psychiatrist stayed with the students in the seminar room the whole time. During the surgeon’s absence she discussed with the students topics regarding communication and interaction on the one hand and moderated the communication between the observing students group and the ward group (for a more comprehensive overview, please see Fig. 1). 16 students took part in the ward round per unit. 12 surgeons of the department of General, Visceral and Transplant Surgery taught the students. One patient was part of the ward round per unit. In total 14 Patients participated in the study course. The patients' diseases were in the field of general surgery.

The technical setup comprised an Apple iPad mini for the actual ward round and a standard laptop with external monitor in the seminar room, both connected via an end-to-end encrypted “Zoom” connection approved by the hospital’s data security office for patient streaming. Due to the bidirectional audio-visual connection, all participants (ward and seminar room) could see and interact with each other. The Students could especially ask questions to the team and the patient.

### Measurement

At the end of the face-to-face teaching seminar students filled in a questionnaire containing questions on their subjective rating of ward round competencies as well as satisfaction with and helpfulness of the course. Additionally, structural and technical aspects of the video-transmitted ward round (e.g. efficiency of the round itself, visibility of the patient or integration of nurse) were assessed. All items ranged on a Likert Scale from 1 (“not at all”) to 5 (“very”). An example item was “how satisfied were you with the general organization of the ward round.”. Furthermore, students rated the surgeon’s empathy in the observed ward round using the student version of the Jefferson Empathy Scale (JES). The JES is a standardizedquestionnaire and consists of 20 items ranging from 1 (“do not agree at all”) to 7 (“completely agree”) [24]. The JES is highly reliable and validated [25]. A value under 80 indicates little empathy while 140 is the highest score indicating a high level of empathy [25]. To cover for the digital aspect, we also asked for technical difficulties experienced in the session and students’ course preference (digital/in-person) using dichotomous answers (yes/no). Furthermore, students could made comments in the qualitative feedback section regarding the teaching course.

### Statistical analysis

Quantitative data were evaluated by using IBM SPSS Statistics version 27. Frequencies, percentages, mean values (M) and standard deviations (SD) were calculated. Data was tested normally distributed by using Kolomogrow Smirnow-test. T-test for dependent samples were used to test for differences in the competencies before and after the course. The level of significance was set to *p *< 0.05. Qualitative feedback was evaluated in thematic content analysis based on Braun & Clark (2006) using Microsoft Excel, and themes in the dataset were identified, analysed and documented [26]. Based on the qualitative data codes and categories were inductively generated and after examining them topics were built [26].

### Ethics

The study was approved by the Ethics Committee of the University Clinic of Tübingen, Project Nr. 272/2021BO2. It was conducted in accordance to the relevant guidelines and regulations and in accordance to the Declaration of Helsinki. Participation was voluntary, and all participants gave their informed written consent. All patients involved were thoroughly informed beforehand and gave their written consent. Patients below the age of 18 or with a limited understanding of German were excluded from participation in the study.

## Results

Overall, students were satisfied with the video-transmitted ward round course rating it with M = 3.54 (SD = 1.22). The Helpfulness of the course was rated with M = 3.92 (SD = 1.01).

In the subjective evaluation students’ ward round competencies rose significantly (*p* < 0.001) from M = 3.0 (SD = 0.77) to M = 3.76 (SD = 0.75) in a pre-post comparison.

Students considered the teacher’s explanation of the lab results as very good (M = 4.73, SD = 0.64). The ward round itself was considered highly efficient (M = 4.64, SD = 0.58). They were also very happy with the explanation of the bedside examination within the ward round (M = 4.77, SD = 0.54). Regarding the interprofessional ward team, the involvement of the nurse was rated relatively high with M = 3.84 (SD = 0.93) reflecting standard operational procedures on the ward. The ward round surgeon was rated as highly empathic by the students with M = 119.05 (SD = 10.09).

**Fig. 2:**
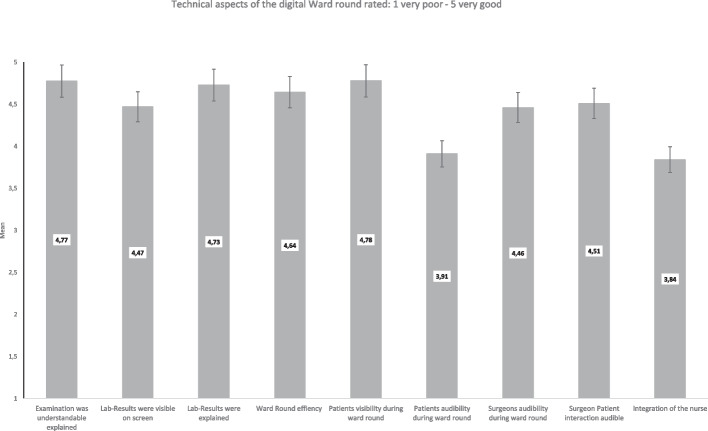
Rating of technical aspects of the video-transmitted ward round. Likert Scale 1–5

Also, the rating of technical aspects was generally positive (for further details, please see Fig. 2); only 8.7% of the students rated technical difficulties with disruptions of the visual connection, reduced sound quality or a break down of the connection as most commonly mentioned.

Rating of the video-transmitted ward round technical aspects.

In general, students appreciated aspects of structure and interprofessional teaching, though still were in favour of face-to-face ward rounds (87.4%) if allowed again. The following thematic topics could be found based on the qualitative analysis: helpful aspects of the video-transmitted ward round, room for improvement, content to be kept after the pandemic and reasons for preference of format (online versus face-to-face). Please see Table 1 for more details.

**Table 1:** Qualitative comments from students regarding video-transmitted ward round

**Helpful Aspects of the video-transmitted ward round**
• General establishment of a structure for a surgical ward round • Realisation of importance to think about a visit beforehand and discuss patient case with other healthcare professionals • Practical case discussions including all findings (lab, x-ray, etc.) • Observations of liaison psychiatrist regarding communication and social interaction aspects of a ward round
**Room for Improvement**
• More Cases during the ward round would be better • Better sound quality • Filmed videos for asynchron online consumation
**Content to be kept after the pandemic**
• Explicit teaching on the topic “ward rounds” • Detailed discussion of the case in advance • Discussion of communicative and interactive aspects • Interdisciplinary teaching approach
**Reasons for preference of format (online versus face-to-face)**
• Online: ◦ Better visibility ◦ Better comprehensibility ◦ Patient not overwhelmed • Face-to-face: ◦ Better interpersonal connection (incl. non-verbal communication) ◦ Easier to ask questions ◦ More “natural”/realistic feeling of a ward round

## Discussion

With this study, we could show that it was feasible to implement a video-transmitted ward round into regular training of undergraduate medical students within a pandemic. The format worked technically, was well-accepted by the target group and also led to a subjective significant rise in surgical ward round competencies of medical students deemed necessary for adequate treatment of surgical inpatients [1].

Due to a structured educational model, explicit teaching of usually implicitly taught skills like communication aspects of a ward round, was implemented and appreciated by students. This was in line with literature stating the importance of communicational aspects of a ward round for patient care and safety as well as the surgeon’s role as a communicator [27–31]. The two-way interaction possibility with all participants involved (interprofessional healthcare team, patient, students) and here especially the possibility to ask questions also made the students feel fully integrated and part of the actual ward round preventing an otherwise commonly observed effect of disengagement all be it in a digital environment [16, 22, 23]. Their appreciation of a more immediate contact is in line with other findings and corroborates well-established concerns of usual surgical ward rounds [20, 21, 32].

The ward round is also a central point for interprofessional care, where crucial aspects of patients are discussed in the team of healthcare professionals [33]. Interprofessional collaboration has been labelled a crucial factor in reducing complications and mortality in patients [34]. In this study, students observed a full integration of the ward nurse and mentioned it as a pivotal learning effect of the session. Raising awareness for the interprofessional aspects within a surgical ward round is a fundamental component of patient-safety [3, 35].

Digital formats for teaching within the pandemic had so far focussed either on the care of actual COVID-19 patients, created specific designs for ward round teaching, or simply let students sit by routine telemedical encounters [17–19, 21]. Although these concepts offer a valuable teaching opportunities, our intention was specifically to preserve the skill of conducting a surgical ward round making use of digital concepts already proven helpful in general bedside teachings [36].

A well-known issue in ward round teaching is, that students lack a structure as also mirrored in our study. Although checklists have shown to be helpful for basic structure, its value to improve the actual communication and interaction on the ward is still unclear [1, 37–39]. We thus used a designated, structured teaching concept, explicitly covering all aspects and key competencies of the ward round cycle – as demanded by research in this field [40, 41]. Especially the interaction and the students’ willingness to ask questions can be improved by such targeted interventions [42].

In this study, the performing surgeon was rated as very empathic. This is particularly important in light of the fact that physicians are eagerly observed by students and act as role models [43]. Awareness to this fact should be risen to all teaching faculty stuff as it is well-proven that positive role models encourage interest in the subject and consequently positively influence career choice [44]. It is also well-known that patient subjectively rate their treatment outcome higher when they consider their surgeon as more empathic [45].

A big issue with digital teaching is the technical component and possible failure of devices or transmission [46]. In this study, the technical side worked pretty well with only a very small group (8.7%) reporting issues. Although most of these were rather minor, it is of utmost importance to guarantee high quality audio-visual set-ups to give a realistic impression and feeling of emersion when performing a video-transmitted ward round. Therefore, the help and support of specialized technical staff should be made available in the faculties particularly if even more digitally advanced elements like augmented or virtual element features are to be integrated [47, 48].

Understandably, students in our study were currently more inclined to have a face-to-face ward round than a digital one which is in line with other findings on bedside teaching settings [20, 49]. However, the dynamics driven by the COVID-19 pandemic may be the catalyst to meticulously scrutinize the positive effects of virtual ward rounds for transfer potential in face-to-face settings combining the best of two worlds and train competent surgeons of the future. Therefore it is relevant to stress that the Students mentioned a better visibility and comprehensibility of the clinical encounters which may be related to the fact that all viewers had the same perspective close to the Patient. In a real world patients room there is usually only a limited space with reduced visibility for some students. We will study this in the future with the help of 3D Videos of ward round situations.

Having one perspective on the situation enabled also a better postexposure discussion as all participants had watched the same footage this corresponds to the helpfulness of videos as a reflexive instrument [50]. Due to the nature of video streams this visualisation was transitory for the future a recorded version of the ward round could help this process even further.

There are some limitations to this study. First, it was within one semester and one medical faculty only which may limit generalisability. Second, we only measured the students’ subjective gain in competency. Future studies should evaluate skills acquired through video-transmitted ward rounds in either real war round settings or simulated sessions objectively. Finally, it was not possible to compare the digital concept to a face-to-face setting due to the Covid-19 restrictions, we only asked the medical students’ perspective. Further research should investigate this comparison to confirm our finding on students’ preference of a face-to-face setting. Furthermore, the patient’s perspective was not assessed in the study. So, we could not compare the patient’s and students’ perspective regarding the physician’ s empathy. Future research should also involve the patient’s perspective. In this study, we decided us against the patient’s perspective as we focused on the feasibility. However, despite these limitations we feel confident that this study contributes significantly to the insights of digital teaching concepts with regards to surgical ward rounds.

## Conclusions

This study showed that it is possible to transfer the powerful tool of ward round teaching successfully into a digital format. Further studies should focus on more detailed and specific components of the ward round and the patients’ perception of such an offer as well as on the transfer potential of digitally helpful elements in a face-to-face ward round structure. Moreover, the communications aspects and the integration of ward nurses should be focused more specifically in future research as, in this study, they might be addressed as implicit manners.

## Data Availability

The datasets used and/or analysed during this study are available from the corresponding author on reasonable request.
